# Differential Evolution of MAGE Genes Based on Expression Pattern and Selection Pressure

**DOI:** 10.1371/journal.pone.0048240

**Published:** 2012-10-25

**Authors:** Qi Zhao, Otavia L. Caballero, Andrew J. G. Simpson, Robert L. Strausberg

**Affiliations:** Ludwig Collaborative Laboratory, Department of Neurosurgery, Johns Hopkins University School of Medicine, Baltimore, Maryland, United States of America; Australian National University, Australia

## Abstract

Starting from publicly-accessible datasets, we have utilized comparative and phylogenetic genome analyses to characterize the evolution of the human MAGE gene family. Our characterization of genomic structures in representative genomes of primates, rodents, carnivora, and macroscelidea indicates that both Type I and Type II MAGE genes have undergone lineage-specific evolution. The restricted expression pattern in germ cells of Type I MAGE orthologs is observed throughout evolutionary history. Unlike Type II MAGEs that have conserved promoter sequences, Type I MAGEs lack promoter conservation, suggesting that epigenetic regulation is a central mechanism for controlling their expression. Codon analysis shows that Type I but not Type II MAGE genes have been under positive selection. The combination of genomic and expression analysis suggests that Type 1 MAGE promoters and genes continue to evolve in the hominin lineage, perhaps towards functional diversification or acquiring additional specific functions, and that selection pressure at codon level is associated with expression spectrum.

## Introduction

The MAGE (**m**elanoma-**a**ssociated anti**ge**n) gene family is composed of genes that all share a homologous MAGE conserved domain of approximately 200 amino acids. In humans the family contains 37 protein-coding genes. Based on their expression patterns, MAGE genes are categorized as either Type I or II. Type I MAGEs are preferentially expressed in developing germ cells and for some of them in placenta, while silent or expressed at low levels in normal adult tissues, but re-expressed in selected tumor types [1], [2] and because of this particular expression pattern, they are classified as members of the cancer/testis (CT) antigen gene family [3], [4]. Type II MAGEs are ubiquitously expressed in normal tissues and cancer cells. Based on their sequence relatedness, MAGE genes have also been assigned to subfamilies. The Type I MAGE subfamilies MAGEA, MAGEB and MAGEC, are all located in clusters on chromosome X. Type II MAGE genes in the subfamilies MAGED, MAGEE, MAGEF, MAGEH, MAGEL and NDN are clustered on chromosome X as well as a few autosomes.

The existence of the MAGE conserved domain can be traced back to the Nse3 gene in yeast (*Saccharomyces cerevisiae*). The Nse3 protein is one of eight subunits in the Smc5–6 protein complex which plays a role in meiosis [5], [6]. MAGE proteins are also found in model organisms such as nematode (*Brugia malayi*), drosophila (*D. melanogaster*) and zebrafish (*Danio rerio*). However, in those systems their functions are not well understood.

Crystal structures of the MAGE conserved domain encoded by human MAGEA4 and NDNL2 (also called MAGEG1) genes show that this domain composed of two winged-helixes motifs may be involved in protein-protein interactions [7]. Indeed, several *in vitro* and *ex vivo* studies suggest involvement of MAGE proteins in transcriptional regulation. MAGEA and MAGEC members have been shown to indirectly interact with TP53 and regulate its stability [8], [9]. Xiao et al identified a role for Type I MAGE in KAP1 and KRAB domain zinc finger transcription factor-based gene repression [10]. Yang and colleagues also demonstrated that several Type I MAGE proteins are able to complex with KAP1 and suppress p53-dependent apoptosis [11]. Recently, Doyle et al showed the interaction of human Type I MAGE proteins with RING domain proteins results in subsequent enhancement of ubiquitin ligase activity [7]. Due to their specific expression in tumors and significant immunogenicity, Type I MAGEs have been widely speculated to play a role in tumorigenesis and cancer progression. A number of clinical studies have associated CT antigen gene expression with more advanced and more aggressive tumors [12], [13], [14]. In contrast, other studies have linked the expression of individual MAGE genes with a better prognosis and longer survival [15], [16], [17]. Thus the role of MAGE genes in cancer, especially Type I MAGEs, is an area of active investigation.

The evolutionary pattern and oncogenic roles of the MAGE family have previously been explored in human and mouse [18], [19], [20]. The availability of additional mammalian genomes provides us the opportunity to revisit the course of evolution of this important gene family. A recent study by Katsura and Satta has reported a thorough analysis on MAGE evolution history. Their focus on the genomic organization of the MAGEA subfamily and nucleotide substitutions between MAGEA3 and MAGEA6 led to the conclusion that negative selection on MAGEA3 and MAGEA6 specifically existed in humans based on interplay with the HLA locus [21]. In this study, we take a different approach by looking at differences within the MAGE gene family composed of Type I and Type II MAGEs based on their expression characteristics in the genomes of eutherians including primates (human, chimpanzee, orangutan and rhesus monkey), rodents (mouse and rat),and carnivores (dog). Our integrative analysis on genomic structures, transcriptomes and codon changes show that different selection forces has been impacting on MAGE genes as determined by different expression spectrums through evolution. Our results provide new insights to the evolutionary history of MAGE genes under different selective pressures currently driving Type I and Type II MAGE evolution and shaping their functions.

## Results

### Signature of MAGE Gene Clusters in Mammals

The current RefSeq dataset (Release 52, Sept. 2011) contains 37 protein-coding human MAGE genes. The MAGE superfamily includes Type I MAGE genes (preferential expression pattern of cancer/testis antigens) and Type II MAGE genes which have a much broader expression pattern (Table 1). We utilized cDNA sequences of the entire human MAGE gene set (*Homo sapiens*, GRCh37/hg19) to search for orthologs and paralogs in the genomes of chimpanzee (*Pan troglodytes*, panTro3), orangutan (*Pongo pygmaeus abelii*, ponAbe2), rhesus monkey (*Macaca mulatta*, rheMac2), mouse (*Mus musculus*, NCBI37), rat (*Rattus* norvegicus, rn4), dog (*Canis familiaris*, Broadv2.0) and elephant (*Loxodonta africana*, loxAfr3) (see Methods). We confirmed known MAGE homologs and identified additional MAGE gene family members encoded in these genomes. Using the human MAGE nomenclature and subfamilies in the RefSeq database, we re-annotated and classified MAGE homologs in the species included in this study. Table 1 summarizes the number of orthologs and paralogs identified in each subfamily. The number of protein-coding MAGE gene is maintained above 30 per genome at least starting with the dog genome belonging to the mammalian order carnivora. Even with the fragmented assembly, at least 10 MAGE genes are discernable in the elephant genome. Details of the gene list and associated reference annotation are provided in Table S1.

**Table 1:** Summary of protein-coding MAGE homologs in each genome.

		Primates				Rodents		Carnivora
Chromosome	Subfamily	Human	Chimpanzee	Orangutan	Rhesus	Rat	Mouse	Dog
Type I								
X	MAGEA	12	10	8	11	8	8	12
X	MAGEB	10	10	10	11	13	11	11
X	MAGEC	3	3	3	4	4	4	2
Type II								
X	MAGED	4	3	3	3	2	2	3
X	MAGEE	2	2	2	2	2	2	0
X	MAGEH	1	1	1	1	1	1	1
X	TRO	1	1	1	1	0	1	0
autosome	MAGEF	1	1	1	1	0	0	1
autosome	MAGEL	1	1	1	1	1	1	1
autosome	NDN	2	2	1	2	3	3	2
Total gene number		37	34	31	37	34	33	33

At the gene level, the MAGE conserved domain is encoded by one exon in all homologs except in the MAGED subfamily. In addition to the ancestral MAGE domain, genes in different subfamilies have acquired additional protein-encoding sequences with specific signatures at the N-terminus that are Proline-rich (MAGEE), Serine-rich (MAGEC) or Glutamine-rich (MAGEL). The acquisition of additional signature sequences is especially prominent in primate genomes. Homologies shared by orthologs are much higher in Type II than Type I MAGE genes (Table S2). For example, there is at least 82% identity in the coding nucleotide sequence between a human MAGE and its ortholog in Type II MAGE genes (MAGEDs, MAGEEs, MAGEF, MAGEL, MAGEH, NDNs and TRO); however the homology could reach just above 60% between an ortholog pair within Type I MAGE subfamily (MAGEAs, MAGEBs or MAGECs). Among all MAGE genes, MAGED is the most conserved subfamily with over 91% identity in the coding sequences between human and dog, the species with further evolutionary distance. The least conserved MAGE genes are members of the MAGEC subfamily.

**Figure 1:**
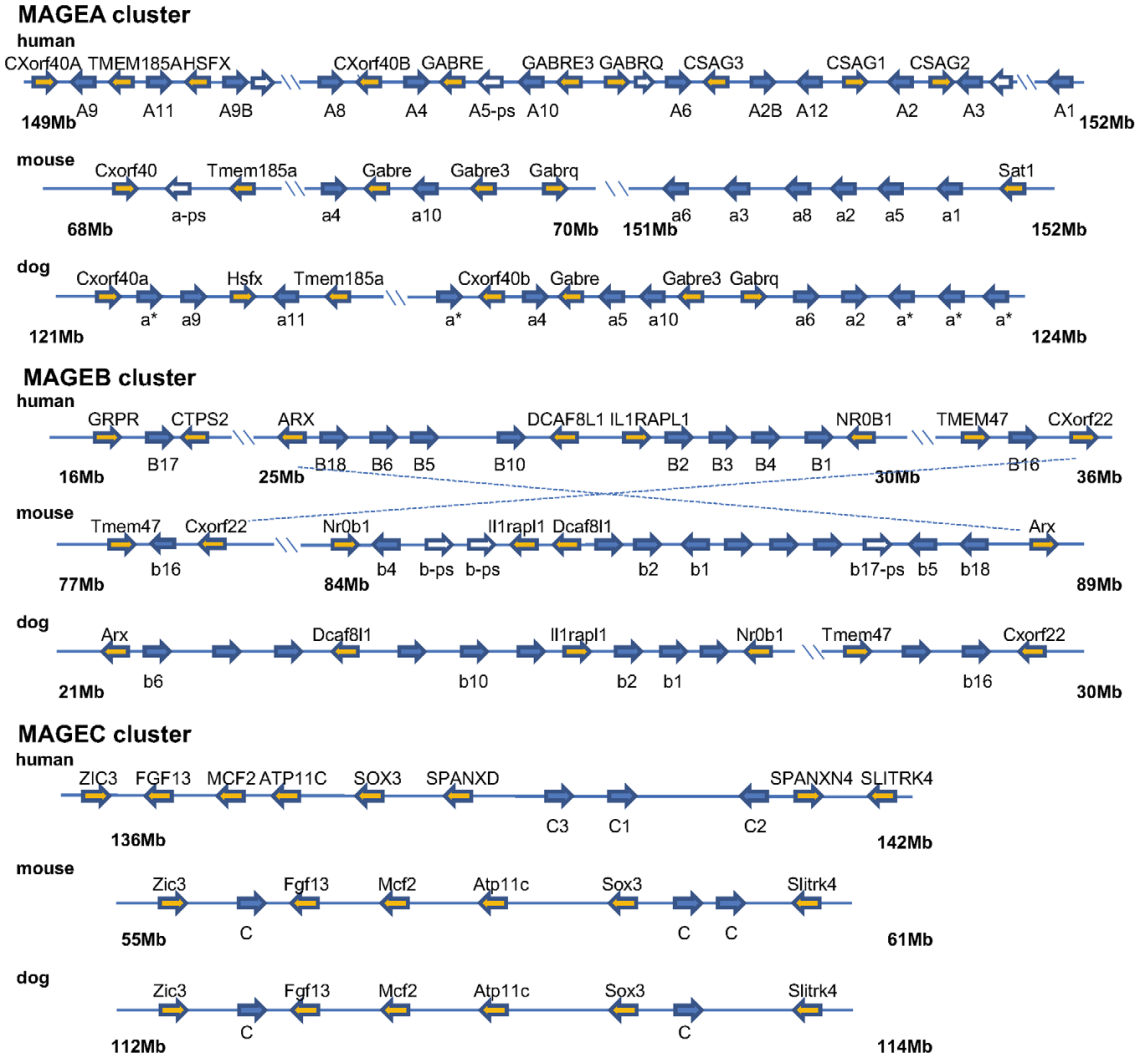
Synteny of Type I MAGE clusters on chromosome X among human, mouse and dog. Approximate coordinates on the X chromosome are labeled under each cluster including MAGEA, MAGEB and MAGEC subfamilies. Arrows represent gene orientation. Anchor genes for synteny other than MAGE genes are represented by orange arrows; copies of MAGE genes are represented by blue arrows; pseudogenes are shown as in white/open arrows. *: gene recruited from unmapped scaffolds in dog genome integrated with phylogeny analysis. Naming of MAGE genes shown are based on RefSeq entries.

Most MAGE genes are located in clusters in all species studied. All of the Type I MAGE gene clusters are located on the X chromosome (Table 1). In human, MAGEA subfamily members cluster at X:q28, MAGEB at X:p21, and MAGEC at X:q26. Type II MAGE gene clusters are also observed on autosomes, as well as the X chromosome. Examination of syntenic blocks containing MAGE genes across genomes indicates that such clusters were preserved in primates, rodents and most likely in carnivores as shown by the dog genome (Figure 1). However, each cluster has undergone a different degree of expansion by duplication or contraction by pseudogenization within each genome analyzed (Figure 1 and Table S1). MAGE pseudogenes are observed in each genome as remnants of homologous sequences remaining at the syntenic locus for both Type I and Type II MAGEs. Clusters formed by Type I MAGE genes vary the most in genomic organization. The mouse and rat genomes carry several pseudogenized MAGEBs and MAGEAs, respectively (Table S1). The structural organization of the dog genome shares similarity with the Type I MAGE clusters in human, but it lacks the Type II MAGEE subfamily. Interestingly, the Type I MAGEA cluster which segregates at one locus on the X chromosome in primates and dog, splits at two X chromosome locations 80 Mb apart in the rodent genomes (Figure 1). This could be the result of an inter-chromosomal recombination event during genome evolution in rodents. Indeed, comparison of the sex chromosome between human and rodents reveals that several rearrangements have occurred in the rodent lineage while the structure of human X chromosome appears to have been remarkably stable since evolved from the common eutherian ancestor [22]. For Type II MAGE genes, species-specific copy number variation is also observed, such as in the MAGED and NDN subfamilies (Table 1). Genomic organization of MAGE genes indicates that MAGE gene family has expanded by genome transposition and local duplication events, undergoing evolution independently in each organism after its split from their phylogenetic ancestor with Type I MAGEs evolving most rapidly.

### Phylogeny Estimation of MAGE Genes

**Figure 2:**
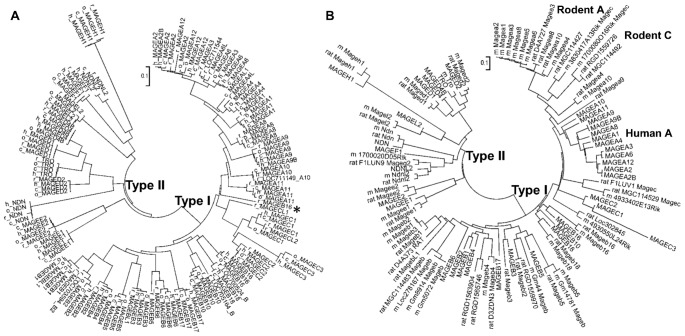
Phylogenetic trees of MAGEs. Two clades are formed: one by Type I MAGE genes; the other by Type II MAGE genes. A, Evolution tree of MAGEs in primates. Gene identifier prefixes with species: h stands for human, c for chimpanzee, o for orangutan and r for rhesus. B, Evolution tree of MAGEs in human and rodents. Rodent gene identifier prefixes with m (stands for mouse) or rat. Genes all in capital letters are from human. The MAGEA subfamily is the most divergent between human and rodents by forming monophyletic clusters of Human A and Rodent A. *, rhesus MAGEB16 however is clustered with MAGEC subfamily (see text). In addition to the conserved MAGEC cluster, new Magec(s) derived from Magea exist in rodents labeled as Rodent C. Trees shown are bootstrap consensus tree with 50% cutoff.

Phylogenetic trees were constructed using neighbor joining method to infer the evolutionary histories of MAGE genes. Coding sequences (CDS) were used for alignments in our analysis. Different substitution measurements produced essentially the same tree topology (see Methods). In the primate MAGE tree (Figure 2A), genes are clustered in two clades: one composed of Type I MAGEs; the other comprising Type II genes. Similar trees formed by two distinct clades are also seen for human-rodents (Figure 2B) and human-dog MAGE genes (Figure S1). Within a clade, each MAGE subfamily forms sub-clade. The trees suggest that Type I MAGE genes diverged early and have evolved independently from the Type II MAGE genes regardless of chromosome location. Two features distinguish the Type I MAGE genes from Type II MAGE genes in the trees: Firstly, the Type I MAGE genes form branches carrying a greater number of leaves/nodes, indicating more rapid expansion. Secondly, branches have a shorter length in the clade formed by Type II MAGEs, indicating slower rate of divergence compared with the Type I MAGE genes (Figure 2). In these trees, nodes (genes) within the same major branch belong to the same subfamily, suggesting appropriate orthologous gene identification and classification. MAGEB16 of rhesus is an exception in that it is clustered with MAGEC branch. However, the nucleotide homology of rhesus MAGEB16 with other primates' MAGEB16 members is at most 78%, which is quite unusual given other orthologous pairs within primates share more than 97% identity. One explanation could be by mis-assembly of rhesus MAGEB16 in the current version of the assembled genome.

The tree topology built on primates MAGEs shows that divergent copies from the same species are not monophyletic, suggesting that members of MAGE genes have originated in early amplification events in the common ancestor of primates and persisted to the present day. MAGEA3/A6 clade seems to have gone through independent duplication in human and rhesus with two closest copies (MAGEA3 and A6) in human and three close homologs in rhesus (Figure 2A). Although only one copy of MAGEA3/A6 homolog has been detected in chimpanzee and orangutan, the possibility that the incomplete genomic assembly status in these two genomes prevents detection of closely related homologues cannot be excluded. A phylogenetic tree constructed from aligned CDSs of human and rodents MAGE genes, however, revealed that MAGEA subfamily members have monophyletic origin in mouse or human (Figure 2B). No pair of mouse and human MAGEA genes possesses a simple 1∶1 orthologous relationship. The same monophyletic topology was also observed for the MAGEA subfamily in the human and dog tree (Figure S1). Inference from the tree topologies suggests that unlike most other MAGE genes that each evolved from its common ancestor since eutherian mammals, the MAGEA cluster has been shaped in primates, rodents, and carnivore lineages independently by local duplication events.

### Variable Natural Selection Pressures on MAGE Genes

We next analyzed the codon changes for evidence of selection pressures [23]. The ratio of nonsynonymous to synonymous substitution rates (ω = Ka/Ks) has been used as an estimate of the selective pressure across the aligned nucleotide sequences that encode proteins. A Ka/Ks ratio close to 1 suggests a neutral evolution for the gene. Whereas a ratio greater than 1 suggests a positive or diversifying selection on the gene, a ratio less than 1 indicates a negative or purifying selection. We first calculated Ka/Ks ratio for each MAGE orthologous pair and found that only Type I MAGEs have a Ka/Ks ratio greater than 1, and this observation exclusively exists in primates, especially between human and chimpanzee. All Type II MAGE genes have a Ka/Ks ratio equal or close to zero (Table S3). When selection pressure varies among amino acid sites, the average Ka/Ks ratio calculated on the entire alignment might not be accurate to infer natural selection forces. We further applied statistical tests on codon changes [24], [25].

**Figure 3:**
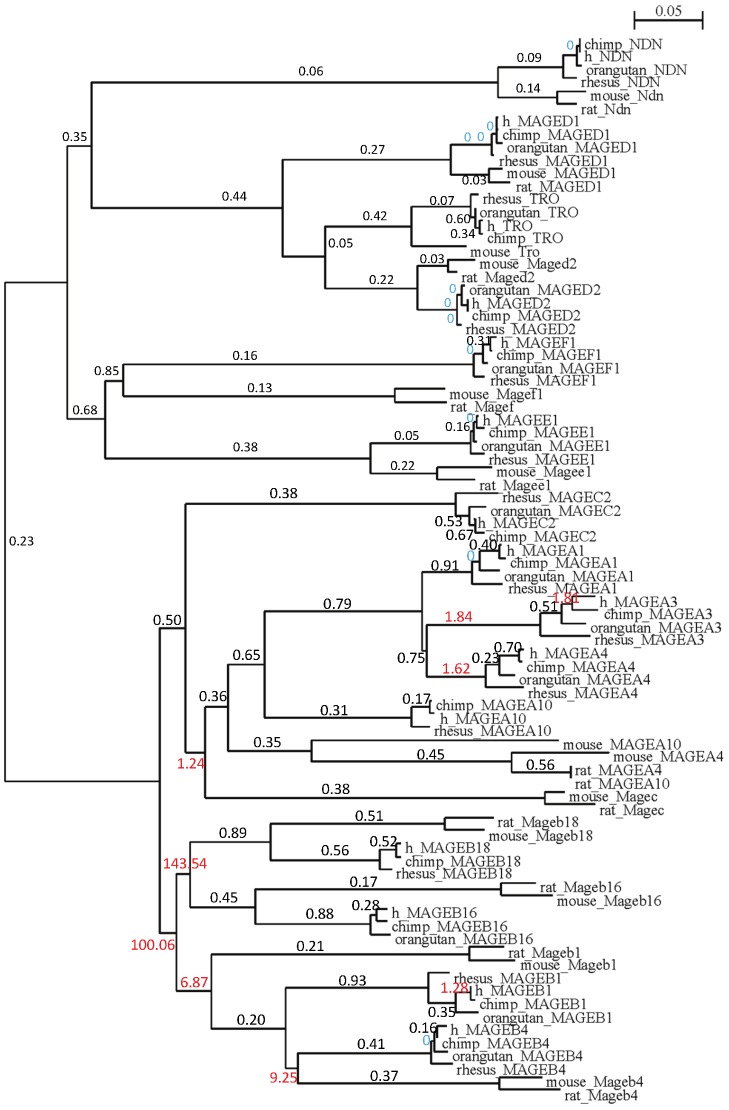
Branch-site test for selection pressure in MAGE genes among lineages. The tree topology displayed was recovered from the maximum likelihood analysis of nucleotide sequences in the codeml test. Numbers labeled on top of each branch are branch-specific ω ratios estimated under free-ratio model. ω>1 is in red font; ω = 0 (stands for ω<0.001) is in blue font.

Ten Type I and six Type II MAGE genes were used to test the selection pressure among individual lineages. The test dataset is composed of codons for the MAGE homology domain, a segment of 585 base pair conserved for all MAGE genes from primates and rodents. Branch-site models in codeml program were applied to this dataset. Likelihood ratio test (LRT) showed that a free-ratio model (independent ω ratio for each branch) is significantly better than the one-ratio model (one ω ratio for all branches) with a *P* value<0.001 (Table 2). We also compared the free-ratio model against the model of neutrality (fix_omega = 1; omega = 1). The LRT further supported the free-ratio model (*P* value<0.001) as shown in Table 2. Under the free-ratio model, the average ω value (ω = 6.18) of branches for Type I MAGEs is significantly higher than that for Type II MAGEs (ω = 0.19), which indicates that Type II MAGEs have evolved under purifying constrains and Type I MAGEs have experienced an elevated rate of non-synonymous changes suggesting positive selection (Figure 3). Branches with ω>1 are exclusively formed by Type I MAGE genes at two evolutionary stages: 1) Emerging or expansion of the MAGEA and MAGEB clusters; 2) In the hominin lineage, a few Type I MAGEs (MAGEA3 and MAGEB1) evolves under positive Darwinian selection. Similar results of purifying selection in Type II branches and positive selection in Type I branches were produced by using branch-site REL model in HyPhy (data not shown).

**Table 2:** Likelihood ratio tests for variable selection pressures among lineages.

Model	Parameter	ω for branches	lnL
One ratio	Model = 0	ω = 0.3088 for all branches	14790.40 (np = 163)
Free ratio	Model = 2	8 branches with ω>1	14645.67 (np = 242)
Neutrality	Fix_omega = 1	9 branches with ω>1	14652.82 (np = 241)

Reject one-ratio model *P*<0.001 (2δ = 289.46, df = 79); reject neutral model *P*<0.001 (2δ = 14.30, df = 1)

**Figure 4:**
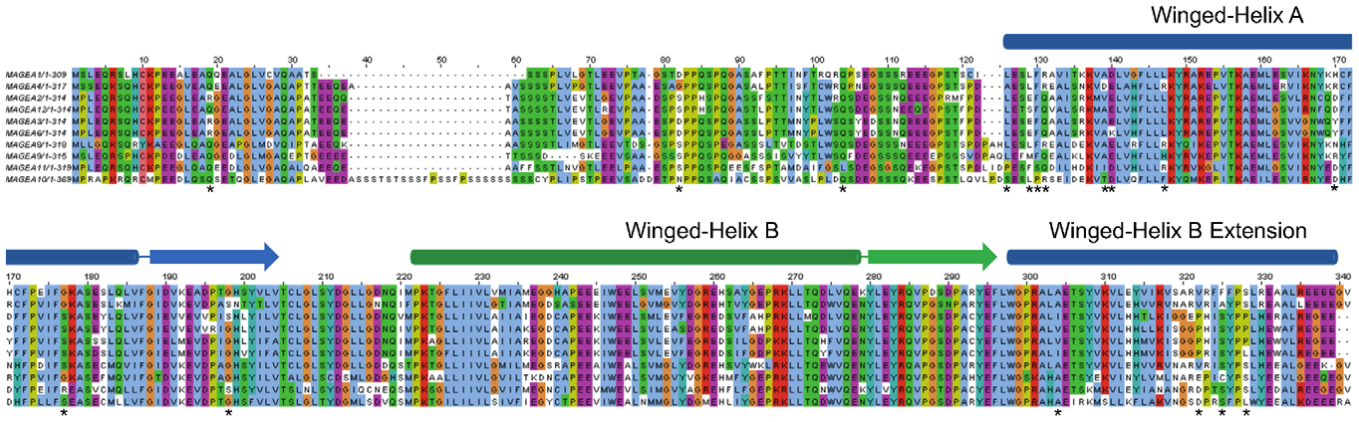
Amino acids estimated under positive selection in genes of the MAGEA subfamily. Codon alignment of human MAGEA genes is used as the reference backbone. *, sites under positive selection predicted by at least two methods as presented in Table 3.

Next, we applied site-specific models to detect positive selection among individual sites. There is a trade-off between more codons to be tested and more taxa/sequences. We first tested MAGE genes within each clade, Type I or Type II MAGE group (Table 3). LRT implemented in the PAML package compares between M1 (NearlyNeutral) and M2 (PositiveSelection) models and between M7 (beta) and M8 (beta& ω>1) models, followed by BEB (Bayes Empirical Bayes) method which identifies specific codon sites under positive selection [26] (Methods). Clearly, positive selection was not detected in Type II MAGE genes. Within Type I MAGEs, positive selection was only detected in human but not in mouse. We further performed the LRT on sub-clades with additional codons to test in the alignments. At this step, we added data from two more primate species (gorilla and gibbon) in order to make the datasets larger with statistical power. Our results suggest that there are a number of codons evolving under strong positive Darwinian selection in genes of the MAGEA subfamily with estimates of ω = 3.43 or ω = 7.17 and LRT test with a *P* value<0.01 (Table 3). Overall, positively selected sites are identified both inside and outside of the MAGE homology domain in MAGEAs (Figure 4). However, such analysis performed on Type II MAGEDs and MAGEEs could not lead to inference of positive selection within these subfamilies. Although no codons under strong positive selection could be detected from estimates for MAGEBs, there is a substantial portion of codons (p1 = 0.23; p1 = 0.73) evolved with a faster non-synonymous change (ω1 = 1.22). We also investigated the signature of selections by applying the FEL and REL methods implemented in the HyPhy package, which incorporate not only nonsynonymous but also synonymous rate variation among codon sites explicitly [27], [28]. Results from FEL and REL show good agreement on positively selected codon sites with those obtained using PAML. As other variables such as sequence composition would introduce systematic bias in model selection [29], the concordant results add confidence to our inference on the estimates.

**Table 3:** Likelihood ratio tests for positive selection on specific sites by PAML and HyPhy.

	# seq	length (nt)	#PSS by FEL	#PSS by REL	M0	M2 (PositiveSelection)	M1 vs. M2 LRT (df = 2)	M8 (beta&ω>1)	M7 vs. M8 LRT (df = 2)	#PSS in M8 by BEB
**Type II MAGEs**										
MAGED_1_4 (primates and rodents)	11	930	N/A	N/A	ω = 0.11; tree length = 1.91	ω1 = 1.00; p1 = 0.017	2δ = 0	ω1 = 1.00; p1 = 0.00001	2δ = 0	N/A
MAGEE_1_2 (primates and rodents)	14	900	1	N/A	ω = 0.19; tree length = 3.85	ω1 = 1.00; p1 = 0.030	2δ = 0	ω1 = 1.76; p1 = 0.018	2δ = 2.38	N/A
All Type II MAGEs (human and chimpanzee)	20	609	N/A	N/A	ω = 0.24; tree length = 10.35	ω1 = 1.00; p1 = 0.043	2δ = 0	ω1 = 4.61; p1 = 0.006	2δ = 2.18	1
**Type I MAGEs**										
MAGEA_1_4 primates subclade*	12	816	2 (78, 105)	2 (78, 105)	ω = 0.68; tree length = 0.91	ω1 = 7.38; p1 = 0.017	*2*δ = 10.78; ***P*** **<0.01**	ω1 = 7.17; p1 = 0.017	*2*δ = 10.74*; * ***P*** **<0.01**	3 (78, 105, 175)
MAGEA_2_3_6_12 primates subclade*	14	912	3 (19, 50, 269)	3 (19, 50, 189, 269)	ω = 0.77; tree length = 1.27	ω1 = 3.55; p1 = 0.078	*2*δ = 11.66; ***P*** **<0.01**	ω1 = 3.43; p1 = 0.083	*2*δ = 11.98; ***P*** **<0.01**	9 (19, 54, 60, 70, 94, 201, 239, 269, 288)
MAGEB_1_2 primates subclade*	12	927	1 (301)	N/A	ω = 0.83; tree length = 1.37	ω1 = 1.22; p1 = 0.73	2δ = 2.06	ω1 = 1.22; p1 = 0.73	2δ = 2.68	4 (55, 78, 235, 301)
MAGEB_16_18 primates subclade	10	966	1 (299)	N/A	ω = 0.64; tree length = 1.54	ω1 = 1.45; p1 = 0.23	2δ = 0.44	ω1 = 1.45; p1 = 0.22	2δ = 1.00	N/A
Human Type I MAGEs*	20	747	6	18	ω = 0.69; tree length = 9.66	ω1 = 1.89; p1 = 0.14	*2*δ = 28.46; ***P*** **<0.001**	ω1 = 1.57; p1 = 0.30	2δ = 53.98; ***P*** **<0.001**	18
Mouse Type I MAGEs	20	603	1	9	ω = 0.40; tree length = 10.46	ω1 = 1.00; p1 = 0.067	2δ = 0.02	ω = 1.49; p1 = 0.054	2δ = 5.48	N/A

#seq, number of nucleotide sequence entries used in the multi-alignments. Length, nucleotide sequence length in alignment. PSS, positive-selected site, predicted by FEL with cutoff of *P*<0.05, REL with cutoff of *P*>0.90 or BEB posterior probability (in M8 by PAML) of *P*>0.90. p1 is proportion of sites in the class with ω>1 and ω1 is the estimate of ω for that class. Numbers in parentheses are codon positions in the alignment. Df, degree of freedom in the LRT. Only significant *P* values in the LRT are presented in bold.*, positive selected sites predicted by different methods are overlapping.

Overall, an average Ka/Ks value of 0.23 has been estimated from a genome-wide collection of 13,454 human-chimpanzee orthologous gene pairs [30]. The average Ka/Ks ratio detected between humans and mice is≈0.2 [31]. The Ka/Ks ratios and statistical testing of the ratios for Type I and Type II MAGEs suggest that they have gone through different selection processes. Type I MAGEs are still undergoing strong adaptive selection in primates, and especially in the huminin lineage. The Type I MAGE genes with a greater than 1 Ka/Ks ratio are those that are most frequently expressed in human testis and tumors such as MAGEA3, A6, and B1. Type II MAGE genes including those on the X chromosome have a Ka/Ks ratio close to zero, indicating that they are under strong purifying constraint.

### Evolution of MAGE Expression and *Cis*-Regulatory Elements

To investigate if the expression of MAGE genes has followed a consistent pattern during evolution, we searched expressed sequence tag (ESTs) libraries in NCBI databases for ESTs corresponding to each gene (see Methods). For the mouse and rat a wide spectrum of defined adult tissues and embryonic developmental stages is represented in databases with more than 4.8 and 1.1 million EST tags, respectively. Orthologs of human Type I MAGE genes have a very similar restricted expression pattern in the adult tissues such as testis and placenta among all species with one difference observed in rodents (Table S4). Interestingly, in mouse, Type I Magea transcripts from the MAGEA subfamily are only detected in oocyte and blastocyst instead of in developing male germ cells and adult testis, even though testis ESTs are well represented in the database. Unfortunately neither blastocyst nor oocyte EST libraries are available for rat to confirm a consistent expression pattern of Magea(s) in rodents. Also interestingly, Mageb16 is expressed in a rat chondrosarcoma cell line of which the corresponding normal cartilage tissue does not express Type I MAGEs. This cell line, rat SRC-JWS cell line, was derived from a tumor that arose spontaneously in a female Sprague-Dawley rat within the lumbar and thoracic vertebra. In addition, messengers of Magea(s) and Mageb(s) have also been detected by microarray experiments performed on mouse melanoma models (GSE29074). These observations suggest that at least some Type I MAGE orthologs are expressed in tumors in other mammals. In rodents, Type II MAGE genes are expressed in many tissue types, consistent with the pattern observed in humans. Moreover, in dog Type I MAGE gene expression is restricted to testis and ovary, similar to expression pattern as seen in human for Type I MAGE, and Type II MAGE expression is wide-spread in other tissues (Table S4). Thus, the restricted or ubiquitous expression for Type I or Type II MAGEs has been inherited at least starting with carnivora. Although the EST data for other primates are limited, the data that do exist suggest this same expression pattern for Type I and Type II MAGEs.

To examine evolutionary patterns for *cis*-regulatory elements or/and promoter regions in Type I and II MAGE genes we aligned sequences flanking 1,000 bp of the transcription start site (TSS) for human MAGE and mouse Mage orthologous gene pairs (see Methods). All Type II MAGE genes have high-scoring segments (HSP) with homology from 60% to 70% identity in the upstream transcription regulatory region between human and mouse orthologs. However, for Type I MAGE genes, homology was rarely detected outside the transcribed exons (data not shown). Introns sequences between orthologous pairs are more conserved in Type II than in Type I MAGEs. We then compared other promoter properties between the two types of MAGE genes in human. We found that all Type I MAGEs do not have a CpG island close to their TSS sites except MAGEA3, MAGEA6 and MAGEB1 with an average CpG dinucleotide count of 26 among the three. In contrast, most Type II MAGE promoters carry a CpG island with an average size of 54 dinucleotides. The observation of difference in CpG island presentation is similar in mouse Mages. In addition, data from transcription factor ChIP-seq of the ENCODE project (see Methods) show that Type I MAGE genes have an average of 5 transcription factors binding at their promoters, substantially less than that of 14 for Type II MAGE genes. These results suggest that epigenetic regulation likely plays an important role in Type I MAGE gene expression. This is consistent with previous studies that have shown promoter methylation playing a role in controlling Type I MAGE expression in human adult tissues and tumors [32], [33].

### Evolution of Expression and Natural Selection in CT Genes

Promoter sequences are subject to natural selection along with coding changes. To investigate if there is a connection between germ line expression and natural selection direction, we extended our Ka/Ks analysis to cancer/testis (CT) genes, which include the Type I MAGE genes. The CT genes encompassing over 150 members in human [34] can be divided into two major subgroups: 1) testis-restricted CTs, which expression is exclusively detected in testis and placenta; 2) testis-selective CTs, which expression is preferentially high in testis but could also be detected at low level in other limited normal tissues such as brain, liver, endocrine system, muscle or/and lung [35].

**Figure 5:**
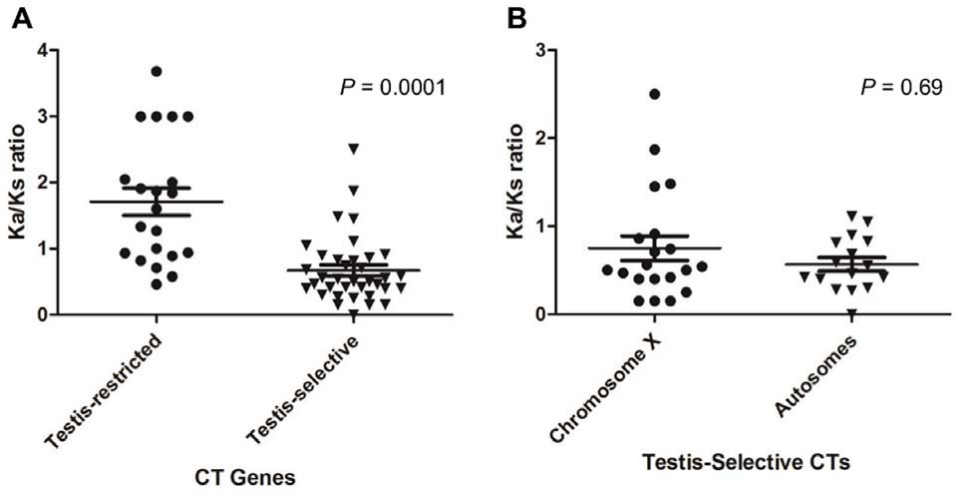
Selection pressure on cancer/testis (CT) antigen genes between human-chimpanzee orthologs. (A), Comparison of Ka/Ks ratios between testis-restricted and testis-selective CT genes. To increase the data entry points, the Ka/Ks ratio is given an arbitrary constant of 3 for Ka/Ks = ∞ which has a Ks = 0. The mean or median of Ka/Ks is significantly higher in the testis-restricted group (*P* = 0.0001 in Mann-Whitney test) than the testis-selected group. (B), Comparison of Ka/Ks ratios for testis-selective genes located on the X chromosome and on autosomes shows no difference (*P* = 0.69 by Mann-Whitney test).

The vast majority of testis-restricted CT genes are located on chromosome X. However, the locations of testis-selective CTs are more balanced between chromosome X and autosomes. Genes in families such as SSX, SPANX, XAGE, CTAGE as well as the Type I MAGEs have representation among both types of CT genes. We further studied the Ka/Ks ratio with human-chimp orthologous pairs between testis-restricted and testis-selective CT gene groups. The Ka/Ks ratio of testis-restricted genes (average ω = 1.69) is significantly higher than those of testis-selective genes (average ω = 0.69) with *P* value = 0.0001 in Mann-Whitney test (Figure 5A and Table S5). However, among testis-selective CTs there is no significant difference of Ka/Ks ratios between genes on chromosome X and genes on autosomes (*P* = 0.69 in Mann-Whitney test in Figure 5B). This result suggests that testis-restricted genes have greater non-synonymous changes, due to at least in part to diversifying selection, compared with testis-selective genes, and it is the expression spectrum, not location on the X chromosome that correlates with and perhaps determines the nature of evolutionary selection for these genes.

## Discussion

### The Origin of Type I and Type II MAGEs

The first evolutionary example of a single MAGE gene (Nse3) is found in the yeast *Saccharomyces cerevisiae*. Based on available genome sequences, a single MAGE is characteristics through the birds. However, starting with the marsupials, two members of MAGE family are observed: in the opossum one is a multi-exon gene located on the X chromosome (XM_001373641), the other is the single-exon gene on chromosome 8 (XM_001365543). Both of these genes exhibit Type II MAGE characteristics.

Based on the local synteny such as nearby PFKFB1 gene and phylogenetic inference, the marsupial MAGE gene on the X chromosome is the ortholog of ancient MAGE gene found in yeast, drosophila, zebrafish and chicken. This copy has expanded locally resulting in the human Type II TRO and MAGED subfamily members clustered at p11.22 on the X chromosome. The MAGE gene located on chromosome 8 of the opossum, likely a processed gene derived from XM_001373641, is the ancestor of Type II MAGEL and perhaps other Type II MAGEs.

**Figure 6:**
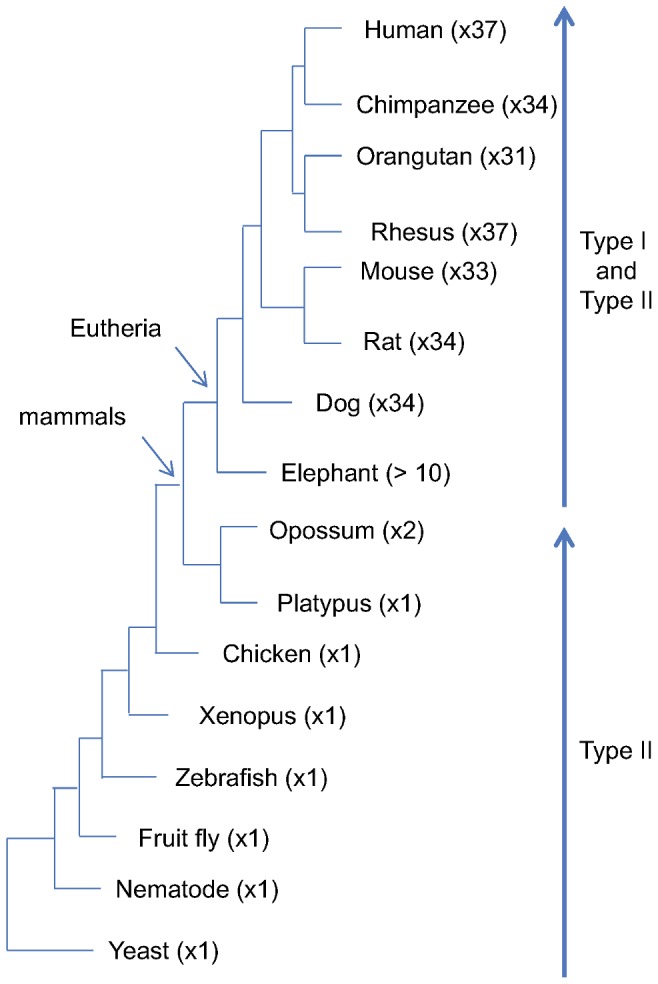
MAGE gene family expansion along evolution. Numbers in brackets are number of total MAGE genes detected in the genome. Type I MAGEs emerged after marsupial.

The emergence of Type I MAGE genes and their expansion is observed only in eutherian mammals after their split from marsupials (Figure 6). Our results are in general agreement with four stages of Type I MAGE evolution recently described by Katsura and Satta [18]: 1) single MAGE gene stage prior to eutherian mammals; 2) subfamily expansion by retrotransposition; 3) expansion by local duplication; 4) reshaping the subfamilies by natural selection.

However, our mining results reveal additional features of MAGE gene evolution. We find that all Type I MAGE subclusters including MAGEA, MAGEB and MAGEC are present throughout eutherian genomes. For example, MAGE genes in each Type I subfamily are present in the elephant genome, the earliest eutherian genome available (Table S1). Especially in the mouse and rat genomes, there is a rodent specific expansion of MAGEC subclusters in addition to the conserved MAGEC subcluster (Figure 2b). There are still unsolved mysteries in the evolutionary history of MAGE. For example, the only MAGE gene in Drosophila is a single-exon gene unlike all other multi-exon single MAGE genes detected in organisms prior to mammals.

Our results indicate that the human and rhesus genomes have the greatest numbers of protein-coding MAGE genes suggesting that the family has been expanding during evolution. Although we observe a reduced number of MAGE genes in the chimpanzee and orangutan genomes, this could result from assembly gaps frequently seen especially on the X chromosome of draft genome assemblies. In the mouse and rat genomes, one active MAGE gene on chromosome 19 and chromosome 1 respectively shows an expression pattern similar to the Type I MAGE genes on the X chromosome. However, they are classified in the Ndn subfamily (Figure 2B) by phylogenetic tree analysis. We suggest that these autosomal singletons result from gene transposition, a mechanism that has previously been shown to generate new genes in mammals [36]. It is worth mentioning that there is a number of naming inconsistencies in the current public databases for MAGE orthologous genes. For example, the only MAGE gene in zebrafish is named Ndnl2, which is the ancestor of the MAGED subfamily. The numerical naming of some subfamily members in the Type I MAGE is not synteny-based across species.

### Differential Evolution of Type I and Type II MAGE Genes

A major focus of this study was to discern patterns of MAGE genes, especially differential selection acting on Type I and II genes. Indeed, our results from Ka/Ks ratio-based statistical analysis shows a major evolutionary difference between Type I and Type II MAGE genes: Type I MAGE genes evolved under positive selection among lineages and among sites whereas Type II MAGE genes evolved under purifying selection. Gene duplication is one of important mechanisms for functional divergence and acquisition of new functions. A pattern of accelerated rate of evolution is commonly seen following gene duplications [37]. However, accelerated changes could be driven by positive Darwinian selection or by relaxation of selective constrains. In the latter case, random fixation of neutral changes could happen in new copies that would eventually lead to functional diversification. Parameter estimates by the lineage-specific tests suggest that positive selection occurred following the translocation of a MAGE copy to the MAGEB locus on chromosome X (Figure 3). Emergence of MAGEA locus on chromosome X was also accompanied by positive selection process (ω>1.2). Then purifying selection returned to these genes with decreased ω (ω<1). Later, positive selection was back to MAGEA and MAGEB genes after local duplication. More interestingly, MAGEA3 and MAGEB1 continued to evolve under positive Darwinian selection at the huminin lineage, suggesting that they acquired adaptive functions in human and chimpanzees. Although there was also increased non-synonymous changes following translocation and gene expansion in Type II MAGEs, the observation was likely due to relaxation of selective constrains with all ω smaller than 1. Later, strong purifying selection has been acting on Type II MAGEs with ω≈0. Overall, evolution of Type II MAGEs shows a pattern of consistent purifying selection. Our site-specific analysis detected codons under positive selection within primate species in genes of Type I but not of Type II MAGEs. Sites under positive selection are mostly found at codons within the conserved MAGE domain which forms the helical loops (Figure 4). Sites outside the MAGE domain are also seen to be subject to positive selection. Comparison of MAGE genes between primates and rodents shows that changes occur in coding regions outside of the MAGE domain, especially in the N terminus. For example, MAGEC1, C3 and E1 have acquired a long stretch of Proline-rich signature at N terminus, in primate genomes only. Proline-rich domains have long been implicated in strengthening protein-protein interactions [38]. Our previous publication has shown that MAGEA and MAGEC genes are frequently mutated in melanoma in their MAGE domain as well as in the N terminus [39].

Proteins involved in mammalian reproduction in male have been the targets of Darwinian selection [40], [41]. It is not surprising that Type I MAGEs which are expressed in reproductive cells or tissues throughout evolution history have been under positive selection. It is speculated that expansion and maintenance of the Type I MAGE gene clusters have important roles in reproduction such as spermatogenesis in primates based on expression location. An interesting finding that a few Type I MAGEA genes continues to evolve under positive selection in the hominin lineage suggests that they have acquired adaptive functions specific to human and chimpanzees. Katsura and Satta provided evidence of potential selection based on the antigenic features of the MAGE proteins, however under negative selection forces [18].

It is intriguing that both Type I and Type II MAGE genes evolved from retrotransposition of the same segment of sequence (the MAGE domain) followed by local expansion on either the X chromosome or autosomes, however they have been under different natural selection forces. In this study, we evaluated evolutionary pressures, pattern of gene expression, the number of gene members within MAGE subfamilies, and potential redundancy of function within MAGE subfamily genes. Most Type II MAGE subfamilies encode only one or a few members that are ubiquitously expressed. We suggest that these subfamilies have already acquired non-redundant and essential functions that now are under purifying selection. However, the positive selection acting upon Type I MAGE genes suggests biologically active individual subfamily members, but with functional redundancy thereby permitting ongoing positive selection toward diversification or acquisition of additional functional characteristics. In order to provide a broader context for the evolutionary patterns of MAGE Type I genes, we extended our analysis to the cancer/testis (CT) gene family, of which Type I MAGE genes are members. One of the features that we sought to address was if the chromosomal location of CT/MAGE genes significantly influences their evolutionary patterns. This is important because previous genome-wide comparison of human and chimpanzee showed that sex chromosomes carry a much faster divergence rate between the two closest primates [30]. Other evolutionary studies have shown that genes on chromosome X are under higher positive selection pressure than those on autosomes in primates [30], [40], [41], [42], [43], [44], [45], [46], [47]. Furthermore, a survey of orthologous cancer-testis (CT) gene pairs in human and chimpanzee indicates that CT genes on chromosome X undergo faster diversifying selection than CTs on autosomes [48]. We did not observe significant differences based on chromosomal location (X chromosome versus autosome). Our results suggest that it is the expression pattern that correlates with and perhaps renders these genes to different forces of natural selection as seen in the two types of MAGE genes. We hypothesize that the local genomic context on each chromosome, including local gene density, repeat density, GC content as well as the recombination rate where evolutionary retrotransposition events have occurred determines the fate of MAGE genes: genes inserted at a gene desert with less genomic sequence complexity become Type I MAGEs and undergo greater expansion. For Type II MAGEs, it is the opposite. Epigenetic controls have been playing an essential role in Type I MAGE expression by default. Indeed, Type I MAGE genes have fewer transcription factor bindings at their promoters than Type II MAGE based on ChIP-seq data. In addition, unlike most Type II MAGEs, which have CpG islands at 5′ promoter region, most of the Type I MAGE genes do not have CpG islands, which have been speculated to protect promoter sequences from being methylated. Studies in human and mice have shown that 80% of CpGs are methylated – but CpGs in CpG islands are usually unmethylated [49]. However, although Type I MAGE gene expression can be turned on by demethylation treatment in tumor cells, that is not the case in cells derived from normal tissue indicating that other mechanisms also exist for regulation of Type I MAGE expression [50].

In summary, our results show that Type I and Type II MAGE genes are both under evolutionary selection. For Type I MAGE genes the selection pressure is positive, and for Type II the selection force is negative. Our analysis of these genes, together with the larger family of cancer/testis antigen genes of which the Type I MAGE genes are members, suggests that the primary ongoing selection is not determined by location on the X chromosome or an autosome. Instead, our results point to the expression patterns of these genes, which relates to acquisition of unique and essential functions, is a major determinant of evolution. For those genes with expression limited to reproductive tissue, in general there is positive selective pressure. Supportive of positive evolutionary pressure, these genes are components of subfamilies with several members, suggesting that there may be at least some redundancy of function, permitting functional diversification or acquisition of new functions in specific members. In contrast, MAGE genes with more ubiquitous expression patterns also have reduced (or no) redundancy. The negative selection pattern observed for these genes argues for their essential cellular functions that require strong evolutionary constraint.

## Methods

### Collection of MAGE Genes

Genome builds used in this study include human (*Homo sapiens*, GRCh37/hg19), chimpanzee (*Pan troglodytes*, panTro3), orangutan (*Pongo pygmaeus abelii*, ponAbe2), rhesus monkey (*Macaca mulatta*, rheMac2), mouse (*Mus musculus*, NCBI37), rat (*Rattus* norvegicus, rn4), dog (*Canis familiaris*, Broadv2.0) and elephant (*Loxodonta africana* draft assembly, Broad/loxAfr3) that are published on UCSC's website or/and by Ensembl. The BLAT tool embedded in the UCSC genome browser was used to search against each genome database using sequences of human MAGE genes as queries. Orthologs are assigned by the mutual best match in the search combined with synteny data in primate genomes; while for rodents and dog, ortholog assignments are guided by chromosome synteny context as well as phylogenetic distance inference. Pseudogenes are defined primarily by two criteria: 1) the gene sequence is identified at syntenic locus based on surrounding markers/genes; 2) no open reading frame – the coding sequence is interrupted by a number of stop codons. Additional evidence for a pseudogene is that it diverges far from its paralogs and orthologs in a phylogenetic tree. Gene identifiers used in Table S1 follow a prioritized order of RefSeq annotation, Ensembl annotation, and computational prediction. In case of computational prediction, a name of MAGE-Like (MAGEL) is assigned to the gene model. For gene models from computation prediction only, EST support is required. Due to the preliminary status of genome assembly for dog and elephant which hinders our effort to build synteny regions, several MAGE genes are found on un-assigned scaffolds. Percent identity reported is for high-scoring segment pairs (HSP) between two sequences by NCBI-BLAST.

### Phylogenetic Analysis

To build a phylogenetic tree, multi-alignments were built by CLUSTALW2 on coding sequence (CDS). Selection of substitution matrix for building a neighbor joining (NJ) tree was estimated by maximum likelihood test implemented in MEGA5 software [51]. Kimura 2-parameter (K2+G) [52] and Tamura-Nei (TN93+G) [53] models are both listed among the top of good measurements, with former being slightly better. Tree topologies generated with the two models are essentially the same. Only NJ trees with K2+G matrix produced in MEGA5 are presented in the figure legend. Bootstrap analysis was performed using a full heuristic approach with 500 replicates.

### Inference of Positive Selection

Codons were aligned by CLUSTALW2 as before with all gaps removed manually without disruption of the reading frame. All insertions are excluded. Deletions are either excluded or if possible filled manually based on consensus. Specifically, any ambiguous alignment at 5′end of the CDS due to short nucleotide repeats or missing sequence was excluded from the subsequent analysis. A pairwise Ka/Ks estimates were performed between orthologous pairs with DnaSP version 5.0 [54]. The significance of difference on Ka/Ks ratios between two groups of CT genes was calculated by non-parametric tests using SPSS software.

For detection of positive selection among individual lineages, codon alignments of MAGE homology domain which is shared by all MAGE genes were used. To limit the size of the test dataset, we picked 8 genes from Type I and 5 genes from Type II to represent the two clades. We applied maximum-likelihood methods implemented in PAML (version 4). For detection of specific codon sites under selection, codons for the entire CDS were aligned for members within a subfamily. A NJ tree topology based on the alignment was provided to codeml. CodonFreq = 2 was used in all analysis. Several branch-site and site-specific models were tested and compared. Estimation of positive selection was inferred from LRT. Individual sites with posterior probabilities >0.90 by Bayes Empirical Bayes (BEB) calculation were reported under NSsites = 8. To show the robustness of the test, we also performed lineage and site-specific tests using HyPhy [55]. Basically, we applied FEL, REL and branch-site REL methods implemented in the HyPhy software and available through the web-based interface Datamonkey (http://www.datamonkey.org/) [56]. All methods are based on maximum-likelihood estimates, REL (random-effects models) assumes that substitution rates across sites can vary according to a gamma distribution and infers the rate at which individual sites evolve; FEL (fixed-effects models) estimates the ratio of nonsynonymous to synonymous substitutions on a site-by-site basis. For both REL and FEL analysis, universal code and HKY85 nucleotide substitution model were used. Due to fragmented assembly information in other primates leading to large alignment gaps, there is limitation on data entries suitable for the positive selection analysis.

### Expression Data Collection and Promoter Characterization

Gene expressions in tissues of chimpanzee, rhesus, mouse, rat and dog were obtained by querying EST libraries deposited at NCBI (data queried on October 01, 2011 from http://www.ncbi.nlm.nih.gov/nucest/). Libraries from pooled tissue sources were ignored. Only ESTs marked with clear library tissue origin were counted. An EST tag matching a MAGE gene with at least 99% identity at 90% length was considered to represent that MAGE gene. Normalization of EST counts was performed by number of matched EST tags per 10,000 ESTs for a given tissue type. Results of normalized EST counts (rounded) are in Table S4. Promoter is defined as 1,000 base pairs flanking the transcription start site (TSS). For genes with alternative start sites, we tested on each TSS. Detection of homologous segments between two promoter sequences was performed with NCBI two-way BLAST. Data for CpG islands and transcription factor ChIP-seq are from ENCODE project obtained from UCSC website (http://genome.ucsc.edu/cgi-bin/hgGateway).

## Supporting Information

Figure S1 **Phylogenetic tree made of human and dog MAGE genes.** Two clades formed by Type I and Type II MAGEs. Dog genes prefix with dog. Bootstrap values over 50% are shown.
(PDF)

Table S1 Mining of MAGE genes across genomes. ps, pseudogene. For models without an annotation, a convention of MAGE-gene-Like is followed.
(XLSX)

Table S2 Nucleotide sequence similarity between MAGE homologs. Percent identity reported is for high-scoring segment pairs (HSP) between two sequences by NCBI-BLAST.
(XLSX)

Table S3 Ka/Ks ratio between orthologous genes. Cells with red font show Ka/Ks>1. Rows highlighted in pink are Type I MAGE genes.
(XLSX)

Table S4 Detection of MAGE gene expression by EST tags deposited in GenBank_EST. Numbers in parentheses are numbers of EST tags matched.
(XLSX)

Table S5 Ka/Ks ratios for MAGE genes between human and chimpanzee orthologous pairs.
(XLSX)
