# Supplementary figures and images for: Differential Evolution of MAGE Genes Based on Expression Pattern and Selection Pressure
Source: PLoS One. 2012 Oct 25;7(10):e48240. doi: 10.1371/journal.pone.0048240 (PMC3484994; doi:10.1371/journal.pone.0048240)

**Figure S1.**

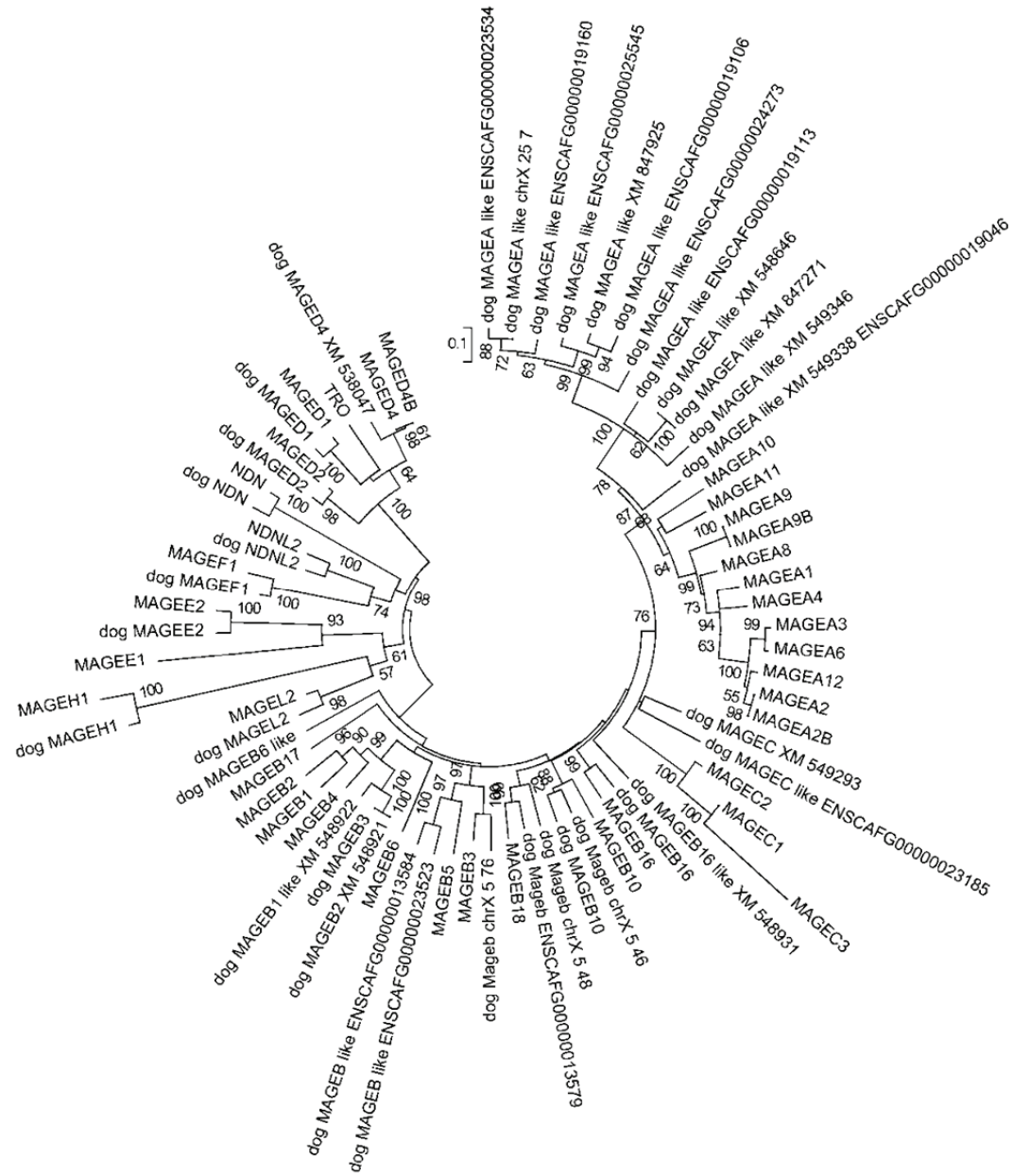

Supplement: Figure S1 — Phylogenetic tree made of human and dog MAGE genes. Two clades formed by Type I and Type II MAGEs. Dog genes prefix with dog. Bootstrap values over 50% are shown. (PDF) [file pone.0048240.s001.pdf]
